# Chitosan Protects Immunosuppressed Mice Against *Cryptosporidium parvum* Infection Through TLR4/STAT1 Signaling Pathways and Gut Microbiota Modulation

**DOI:** 10.3389/fimmu.2021.784683

**Published:** 2022-01-14

**Authors:** Sajid Ur Rahman, Haiyan Gong, Rongsheng Mi, Yan Huang, Xiangan Han, Zhaoguo Chen

**Affiliations:** Key Laboratory of Animal Parasitology of Ministry of Agriculture, Laboratory of Quality and Safety Risk Assessment for Animal Products on Biohazards (Shanghai) of Ministry of Agriculture, Shanghai Veterinary Research Institute, Chinese Academy of Agricultural Sciences, Shanghai, China

**Keywords:** *Cryptosporidium parvum*, chitosan, gut microbiota, TLR4/STAT1 signaling pathways, Bacteroidetes

## Abstract

*Cryptosporidium parvum* infection is very common in infants, immunocompromised patients, or in young ruminants, and chitosan supplementation exhibits beneficial effects against the infection caused by *C. parvum*. This study investigated whether chitosan supplementation modulates the gut microbiota and mediates the TLR4/STAT1 signaling pathways and related cytokines to attenuate *C. parvum* infection in immunosuppressed mice. Immunosuppressed C57BL/6 mice were divided into five treatment groups. The unchallenged mice received a basal diet (control), and three groups of mice challenged with 1 × 10^6^ C*. parvum* received a basal diet, a diet supplemented with 50 mg/kg/day paromomycin, and 1 mg/kg/day chitosan, and unchallenged mice treated with 1 mg/kg/day chitosan. Chitosan supplementation regulated serum biochemical indices and significantly (*p <* 0.01) reduced *C. parvum* oocyst excretion in infected mice treated with chitosan compared with the infected mice that received no treatment. Chitosan-fed infected mice showed significantly (*p <* 0.01) decreased mRNA expression levels of interferon-gamma (IFN-γ) and tumor necrosis factor-α (TNF-α) compared to infected mice that received no treatment. Chitosan significantly inhibited TLR4 and upregulated STAT1 protein expression (*p <* 0.01) in *C. parvum*-infected mice. 16S rRNA sequencing analysis revealed that chitosan supplementation increased the relative abundance of Bacteroidetes/*Bacteroides*, while that of Proteobacteria, Tenericutes, Defferribacteres, and Firmicutes decreased (*p <* 0.05). Overall, the findings revealed that chitosan supplementation can ameliorate *C. parvum* infection by remodeling the composition of the gut microbiota of mice, leading to mediated STAT1/TLR4 up- and downregulation and decreased production of IFN-γ and TNF-α, and these changes resulted in better resolution and control of *C. parvum* infection.

## Introduction

The mammalian gut consists of substantial bacterial communities (microbiota) of 500–1,000 species with a total number of nearly 100 trillion (1, 2). These large microbial communities play an important role in energy production for the host, protection against various pathogens, and shaping of the intestinal epithelium (3, 4). The microbiota also contributes to the regulation of host immune responses (5), and any disturbance or invasion and proliferation of some pathogenic organisms in the gut during the normal process alters the microbial composition, which can affect the health of the host. Diet is one of the most important influencing factors that directly affect microbial communities and their normal function (6). Therefore, the use of nutritional interventions and plans to alter gut microbial communities is a smart way to promote host health.

Chitosan is a nontoxic polysaccharide obtained mainly from crustaceans and shrimps (7). Due to its multiple beneficial effects, chitosan is used in various fields including cosmetics, agriculture, food packaging, and pharmaceutical industries (8, 9). The intrinsic factors of chitosan are mainly responsible for the antimicrobial activity of chitosan (8). Numerous studies have been conducted to investigate the therapeutic effects of chitosan against various microbes and its relationship with various applications, including its nanoparticles, which are more effective for health (10). Recent studies have shown that chitosan nanoparticles (Cs NPs) are an effective agent against fungal, protozoan, and bacterial diseases and are commonly used as transporters for drug delivery (11, 12). These nanoparticles have also been shown to be highly effective for public health, as they are effective against *Toxoplasma*, *Trypanosoma*, *Plasmodium*, and *Leishmania* both *in vivo* and *in vitro* (13–16). Recent studies have also confirmed the anti-*Cryptosporidium* efficacy of chitosan and its nanoparticles in CD-1 outbred mice and HCT-8 and Caco-2 cell lines (17, 18), but the protective mechanisms remain to be elucidated.

Chitosan is produced by DE acetylation of chitin, which happens to be linked to N-acetyl-d-glucosamine and β-(1-4)-linked d-glucosamine (19). Previous studies have shown that chitosan improves the gut microbiota by decreasing the diversity of Firmicutes and increasing the *Bacteroides* population, and efficiently promoting the growth of probiotics such as *Bifidobacterium* and *Lactobacillus* (20). Moreover, studies reported that chitosan supplementation significantly increased the abundance of *Escherichia* in rats (21). However, in weaned pigs, chitosan supplementation decreased the relative abundance of *Lactobacillus* (22). Similarly, chitosan improved lipid metabolism, alleviated metabolic disorders, and positively affected the gut microbiota in rats (21). Studies also reported that chitosan serves as a regulator of immune responses by modulating the expression of various inflammatory cytokines such as Th1 and Th2 (23, 24). Protozoa residing in the gut can alter the composition of the microbiome (25), and these changes can have significant effects on gut homeostasis and host immunity (26). However, the details of the effects and their mechanisms of action are still not clear. Therefore, in the present study, it was hypothesized that chitosan supplementation could modulate the gut microbiota and mediate the activation of some signaling pathways and inflammatory responses in mice challenged with *Cryptosporidium parvum*. To explore the protective mechanisms of chitosan supplementation against *C. parvum* invasion, we examined the microbial communities and inflammatory cytokines and associated signaling pathways in immunosuppressed juvenile mice infected with *C. parvum* oocysts.

## Material and Methods

### Ethical Approval

The experimental protocol was approved by the Animal Ethical Committee of Shanghai Veterinary Research Institute, Chinese Academy of Agricultural Sciences (SV-20201231-03).

### Diet and Parasite

Chitosan powder was purchased from Sigma Aldrich (St. Louis, MO, USA) with an average molecular weight of 100 kDa, a degree of deacetylation of 95%, and high purity free of endotoxins. The chitosan was dissolved in a 1% (w/v) aqueous acetic acid solution with stirring to achieve complete dispersion of the product. ddH_2_O was gradually added while stirring until the solution was completely dissolved. The pH of the final solution was then adjusted to 7 with 1 mol/L NaOH.

In order to generate additional oocysts, neonatal lambs were infected with *C. parvum* Iowa isolate oocysts orally in milk. A similar procedure has been previously performed in neonatal calves in our laboratory (27). After the onset of diarrhea in neonatal lambs, the fresh *C. parvum* oocysts were separated from the feces using the same method as previously performed (28). The fresh *C. parvum* oocysts were stored at 4°C in 2.5% potassium dichromate until use.

### Animals, Experimental Design, and Sampling

One hundred sixty specific pathogen-free (SPF) C57BL/6 juvenile mice (approximately 3 weeks old) purchased from Jie Si Jie Laboratory Animal Co., Ltd. (Shanghai, China) were randomly divided into 5 groups (n = 32 per group). The groups included normal control (N.C), infected + untreated (Inf.Unt), infected + paromomycin treated (Inf.Par) (50 mg/kg/day), infected + chitosan treated (Inf.Chit) (1 mg/kg/day of chitosan), and uninfected + chitosan treated groups (Uni.Chit) (1 mg/kg/day of chitosan). Before infection, all mice received intraperitoneal (IP) injection of dexamethasone (Solarbio Life Sciences) at a dosage of 0.80 mg/mouse/day for seven times to make them immunosuppressed as performed previously (29). After immunosuppression, the mice were orally infected with 1 × 10^6^ C*. parvum* oocysts suspended in ddH_2_O. Treatment drugs were administered orally to the mice on the day before infection and 1, 4, 7, 10, and 13 days post-infection (DPI). Bodyweight (BW) was measured each time before the drug administration using a digital balance. Fresh fecal samples were collected daily from the rectum to quantify parasite oocyst excretion and analyze the gut microbiota. Four mice from each group were sacrificed at one-day intervals. The contents of the ileum were removed and immediately stored at −80°C until RNA and protein extraction.

### Fecal *C. parvum* Oocysts Shedding

The collected mice feces were mixed in 35 ml ddH_2_O and centrifuged at 2,800 rpm for 10 min. Sheather’s sucrose solution was added to the collected sediments, and after complete mixing, centrifuged again at 1,500 rpm for 10 min. After washing several times with ddH_2_O, the final sediment was suspended in 1 ml of ddH_2_O and stored at 4°C until further analysis. Genomic DNA for the determination of *C. parvum* oocysts in feces was extracted using the FastDNA™ SPIN Kit for Soil according to the manufacturer’s instructions (MP, USA). Fecal excretion of *C. parvum* oocysts was measured daily by real-time PCR assay using the primers (JVAF: 5′-ATGACGGGTAACGGGGAAT-3′; JVAR: 5′CCAATTACAAAACCAAAAAGTCC-3) and probe (JVAP18S: 5′-Cy5-CGCGCCTGCTGCCTTCCTTAGATG-BHQ-3) targeting 18S rRNA used previously in our laboratory (27). Briefly, the 20 μl reaction mixture contained 6 μl ddH2O, 2 μl template DNA, 10 μl 2× Premix Ex Taq (TaKaRa), 0.2 μl Rox Reference Dye II, 0.8 μl of each primer, and 0.2 μl probe at the final concentration. The major steps of real-time PCR included denaturation at 95°C for 5 min, followed by 40 cycles of denaturation at 94°C for 10 s. Annealing was performed at 55°C for 30 s and extension at 72°C for 20 s. Amplification reactions were performed on an Applied Bio systems 7500 FAST instrument (Applied Biosystems, USA). To obtain an accurate result, all samples were run three times.

The following formulae were applied to calculate the overall oocysts reduction rate:


(1) Reduction rate %=(infected group−drug treated group)÷infected group×100=final reduction rate

### Analysis of Serum Biochemical Parameters

Blood samples of 400–500 μl were collected from the orbital sinus at 1-day intervals (i.e., days 1, 3, 5, 7, 9, 11, 13, and 15 post-infection) after the mice had been fasted for at least 6 h. However, for experimental purposes, we chose only days 1, 5, and 15 post-infection because D1PI was the onset, D5PI was the peak of *C. parvum* infection, and D15PI was the end of experimental period. Samples were collected under short-term anesthesia with isoflurane by inhalation. The blood samples were centrifuged at 3,500 rpm for 10 min at room temperature to obtain the serum. The collected serum was stored at −80°C until biochemical analysis. The concentrations of creatinine, urea, albumin, total protein, glucose, alkaline phosphatase (ALP), alanine aminotransferase (ALT), and aspartate aminotransferase (AST) in the sera of the mice were determined using an automated analyzer.

### DNA Extraction and 16S rRNA Sequencing Analysis of Gut Microbiota

For microbial diversity analysis, we used third-generation full-length sequencing technology based on the PacBio sequencing platform. Fecal genomic DNA of C57BL/6 was extracted with cetyltrimethylammonium bromide and sodium dodecyl sulfate (CTAB/SDS), and DNA purity and concentration were confirmed by 1% agarose gel electrophoresis. PCR amplifications were performed using 16S primer 27F (5′- ACTTCGTACTTACGTAAT-3′) and 1492R (5′-TGCATTATTAGCATTAA-3′) primer set. PCR was performed in 30-μl reaction mixtures with 15 μl Phusion. The mixtures consisted of 0.2 μM of each primer and 10 ng of template DNA. The protocol for PCR consisted of denaturation at 98°C for 1 min, 30 cycles of 98°C for 10 s, 50°C for 30 s, and 72°C for 60 s, and a final elongation at 72°C for 5 min. Sequencing libraries were prepared and index-coded using NEB Next Ultra DNA Library Prep Kit for Illumina (NEB, USA) according to the manufacturer’s protocol. The quality of the sequencing libraries was measured using Agilent Bioanalyzer 2100 systems and Qubit@ 2.0 fluorometer (Life Technologies, CA, USA). Finally, the library was sequenced on an Ion S5TM XL platform with 400 bp/600 bp single-end reads.

The software tool Fast Length Adjustment of Short reads (FLASH, v1.2.7) was used to combine the original DNA fragments. The obtained sequences were analyzed using Quantitative Insights in Microbial Ecology (QIIME, v1.6.0) software after each sequence was assigned to samples using barcoded primers. Adapters and primers were truncated, and all sequences were quality filtered, denoised, merged, and chimeras removed using Divisive Amplicon Denoising Algorithm 2 (DADA2). The single-end reads were combined and clustered into amplicon sequence variants (ASVs) with 99% OTUs reference sequences using QIIME 2. Taxonomic ranks were assigned to the typical sequences using the Classify-Sklearn Naive Bayes Taxonomy Classifier. A representative ASV for each group on each day was visualized in a Venn diagram using the software tool R (v3.1.1). In each sample, the relative richness of ASVs was recorded using principal component analysis (PCA). Functional annotation or differences in the abundances of each taxonomy between the two groups were visualized using Systems-Theoretic Accident Model and Processes (STAMP) analysis. The taxonomy (phylum, class, order, family, genus, and species) or relative abundances of ASVs were summarized in figures or tables. Species cluster analysis based on the abundance of each species was presented in a heat map, and species in all samples whose richness was <0.5% were classified as “other.” The tags with the highest richness of each genus were selected as the corresponding genus-specific sequences, and a genus-level phylogenetic tree was constructed using the QIIME software tool. Diversity for each sample was visualized using a series of indices including alpha diversity metrics such as Chao1, ACE, Shannon, Simpson, and beta diversity weighted UniFrac were estimated using the diversity plugin, and all figures were created using R (v3.1.1).

### Detection of Intestinal Cytokine Expression by qPCR

Total RNA from intestinal tissues at day 1, 5, and 15 post-infection was extracted with Trizol reagent (Invitrogen, Carlsbad, CA). The mRNA expression of cytokines including IFN-γ and TNF-α was determined by qPCR. Briefly, after RNA extraction, Invitrogen Reverse Transcription Kit Superscript III and Reverse Transcription System (Invitrogen) were used to reverse transcribe total RNA. Gene expression was measured with an ABI 7900 Fast Real-Time PCR system thermocycler (Applied Biosystems, USA) using SYBR Green Kit (TaKaRa). The mRNA gene expression levels were calculated according to the formula 2(^−ΔCt^), where ΔCt for each sample = (Ct_target gene_ − Ct_GAPDH_). Glyceraldehyde-3-phosphate dehydrogenase (*GAPDH*) gene served as the housekeeping gene. Twenty-microliter amplification system was prepared according to the manufacturer’s instructions. A targeted and a reference gene primer were utilized to amplify the samples. The primer sequences for each gene are listed in **Table S1** of the supplemental data.

### Western Blot Analysis

Intestinal samples (ileum) collected from mice on days 1, 5, and 15 post-infection were washed twice with cold phosphate-buffered saline (PBS) and centrifuged at 800 × g for 10 min. Total protein from the tissue samples was extracted using a tissue protein extraction kit (X-Y Biotechnology, Shanghai, China) according to the manufacturer’s protocol. Protein concentrations were measured using the BCA assay. SDS -PAGE were used to separate proteins and transferred to polyvinylidene difluoride (PVDF) membranes. After transfer, the membranes were rinsed with phosphate-buffered saline (PBST) for 5 min on a shaker and then blocked with 5% skim milk containing 0.5% Tween-20 and 100 μl Tris-buffered saline for 1 h at room temperature. The membranes were then incubated with the primary antibody of STAT1, TLR4, and β-actin (Proteintech, Co., Ltd.) for 2 h in a shaker. All membranes were then washed three times (5 min each) and incubated again with the secondary antibody for 1 h. Protein bands were photographed using a ChemiDocTM Touch Imaging System (Bio-Rad, Hercules, CA, USA).

### Statistical Analysis

The obtained data were analyzed using Statistical Package for the Social Sciences (SPSS) v27.0 statistical software (SPSS Inc., Chicago, IL, USA) and Graph Pad Prism, v9.0 (Grappa Software, San Diego, CA, USA). The analyzed data were presented as the mean ± SD. Statistical differences were determined using one-way analysis of variance. Protein band intensity for Western blot (WB) was measured using Quantity one software (Bio-Rad). When differences were *p <* 0.05, values were considered statistically significant, and *p <* 0.01 was considered highly significant difference.

## Results

### Chitosan Supplementation Increased BW and Improved the Clinical Symptoms in Mice Infected With *C. parvum*

The mice in the N.C and Uni.Chit treated groups grew normally during the experimental period. However, the mice in the Inf.Unt group showed a significant (*p <* 0.01) reduction in BW during the experimental period (**Table S2**). Detailed trend in BW gain is presented in **Table S2** of the supplemental data. Other clinical signs, mainly sluggish movement, ruffled fur, lack of movement, and loss of appetite, were also observed in mice infected with *C. parvum* and not receiving any treatment compared to the mice treated with chitosan. In contrast, the above signs in the Inf.Par and Inf.Chit groups of mice improved after a few days of drug administration, and they began to drink water and eat properly. No animals died during the experimental period.

### Chitosan Reduced Oocyst Excretion

**Figure 1:**
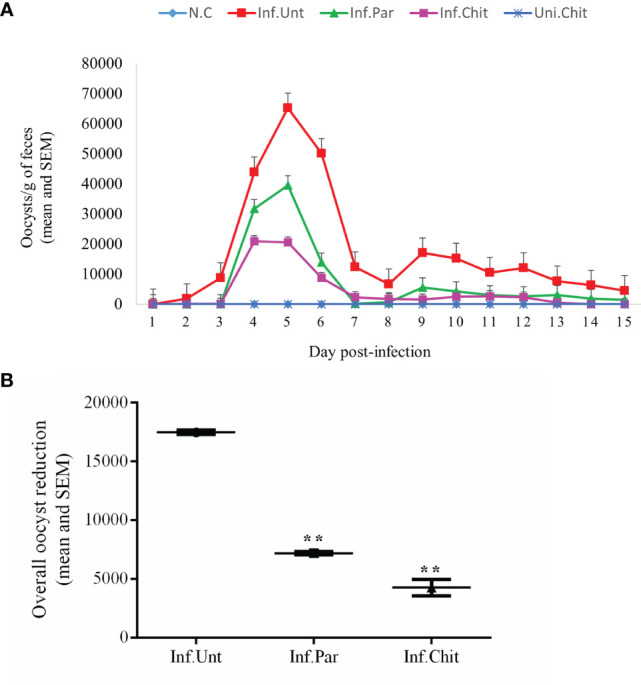
*Cryptosporidium parvum* oocyst shedding pattern. **(A)** Infection intensity in different groups during treatment days. *C*. *parvum* infection was established by oral gavage of ∼10^6^ *C*. *parvum* oocysts followed by oral treatment with the indicated dose of chitosan or paromomycin (positive control). Lines and dosing regimen are color coded as follows: blue line (normal control); red line (infected + untreated); green line (infected + paromomycin treated 50 mg/kg/day); purple line (infected + chitosan treated 1 mg/kg/day); darker blue line (uninfected + chitosan treated 1 mg/kg/day). **(B)** Overall oocyst reduction rate between Inf.Unt, Inf.Par, and Inf.Chit treatment groups. Data are the mean ± SEM of three independent tests. **p < 0.01 statistical difference comparison of the Inf.Unt group with Inf.Par and Inf.Chit groups.

The *C. parvum* oocyst excretion in mice from day 1 to 15 post-infection is shown in **Figure 1**. Since the standard curve was generated using the 18S rRNA sequence, 20 gene copies corresponded to one oocyst. The result showed that the mice excreted the greatest number of oocysts at D5PI, and this was the day of peak infection. At D5PI, the excretion of *C. parvum* oocysts in Inf.Par (39,553.75 ± 28.59) and Inf.Chit (20,537.37 ± 44.38) showed a remarkable decrease in oocyst excretion with a time-dependent pattern observed in the Inf.Unt group (65,218.60 ± 14.17) (*p <* 0.01). As shown in **Figure 1A**, the oocyst excretion in Inf.Par and Inf.Chit groups compared to the Inf.Unt group showed a remarkable decrease from sixth day onward. Remarkably, *C. parvum* oocyst excretion did not cease in the Inf.Unt group even on the last day of treatment (D15PI), whereas no *C. parvum* oocysts were found in Inf.Chit group after D13PI. Overall, a significant decrease (*p <* 0.01) of 58.82% (7,198.47 ± 121 oocysts) in Inf.Par and 75.54% (4,275.90 ± 703 oocysts) in Inf.Chit group was observed when compared to Inf.Unt group with (17,481.88 ± 194) oocysts during the whole experimental period (*p <* 0.01) (**Figure 1B**).

### Chitosan Regulate Biochemical Indices

The changes in the biochemical indices of the mice at different DPIs and during the treatment period are shown in **Table 1**. On the 5th and 15th day of treatment, a significant decrease in albumin level was observed, while total protein level was significantly increased in Inf.Unt group as compared to other groups (*p <* 0.05). On the other hand, ALP, ALT, and AST levels were significantly increased (*p <* 0.05 or *p <* 0.01) in the Inf.Unt group compared with N.C, Inf.Par, Inf.Chit, and Uni.Chit groups. In addition, creatinine levels at day 5 and 15 post-infection decreased significantly (*p <* 0.05 or *p <* 0.01) in the Inf.Unt group compared with the other groups. Urea level during day 1 and 15 post-infection showed an increasing trend, but at D15PI, it was significantly decreased (*p <* 0.05 or *p <* 0.01) in the Inf.Unt group compared with the other groups. Significant changes (*p <* 0.05 or *p <* 0.01) between N.C and infection groups were observed in glucose level at day 5 and 15 post-infection.

**Table 1:** Changes in serum biochemical parameters of *C. parvum*-infected mice following chitosan treatment.

Parameters	Groups	Day 1	Day 5	Day 15
	N.C	2.73 ± 0.11	2.79 ± 0.04	2.78 ± 0.02
	Inf.Unt	2.58 ± 0.15	2.57 ± 0.07^*^	2.60 ± 0.04^*^
Albumin (g/dl)	Inf.Par	2.75 ± 0.10	2.78 ± 0.08	2.76 ± 0.03^#^
	Inf.Chit	2.74 ± 0.06	2.83 ± 0.03	2.73 ± 0.04^#^
	Uni.Chit	2.72 ± 0.08	2.87 ± 0.02^#^	2.76 ± 0.03^##^
	N.C	547.45 ± 0.39	514.59 ± 0.03	368.51 ± 0.45
	Inf.Unt	569.35 ± 0.17^*^	486.22 ± 0.02**	446.47 ± 0.34^**^
ALP (U/L)	Inf.Par	559.22 ± 0.19^#^	528.22 ± 0.19^##^	369.44 ± 0.14^##^
	Inf.Chit	546.36 ± 0.06^#^	561.54 ± 0.46^##^	395.40 ± 0.35^##^
	Uni.Chit	545.34 ± 2.66^#^	517.60 ± 0.35^##^	371.52 ± 0.25^##^
	N.C	41.36 ± 1.2	42.08 ± 2.61	38.45 ± 0.85
	Inf.Unt	42.46 ± 0.43	107.26 ± 2.64^**^	88.81 ± 2.09^**^
ALT (U/L)	Inf.Par	43.96 ± 2.08	65.96 ± 2.17^##^	46.76 ± 0.55^##^
	Inf.Chit	39.29 ± 0.98	54.47 ± 2.65^##^	36.81 ± 1.05^##^
	Uni.Chit	37.92 ± 0.42	37.98 ± 1.14^##^	36.46 ± 0.58^##^
	N.C	78.13 ± 0.13	81.08 ± 0.12	115.39 ± 0.45
	Inf.Unt	98.47 ± 0.26^*^	112.48 ± 0.26^**^	150.54 ± 0.02^**^
AST (U/L)	Inf.Par	87.40 ± 0.42^#^	99.72 ± 0.32^##^	127.21 ± 0.18^#^
	Inf.Chit	83.76 ± 0.39^#^	98.39 ± 0.85^##^	115.55 ± 0.25^##^
	Uni.Chit	79.72 ± 0.36^##^	98.30 ± 1.02^##^	115.52 ± 0.68^##^
	N.C	0.66 ± 0.03	0.87 ± 0.04	0.89 ± 0.04
	Inf.Unt	0.97 ± 0.07^**^	0.75 ± 0.03^*^	0.65 ± 0.04^**^
Creatinine (mg/dl)	Inf.Par	0.71 ± 0.05^##^	0.84 ± 0.02^#^	1.03 ± 0.05^##^
	Inf.Chit	0.79 ± 0.05^##^	0.88 ± 0.03^#^	0.94 ± 0.02^##^
	Uni.Chit	0.62 ± 0.03^##^	0.90 ± 0.04^##^	0.96 ± 0.02^##^
	N.C	105.45 ± 0.04	83.21 ± 0.18	135.45 ± 0.05
	Inf.Unt	105.47 ± 0.12	72.22 ± 0.15^*^	96.49 ± 0.07^**^
Glucose (mg/dl)	Inf.Par	105.39 ± 0.93	93.14 ± 0.12^##^	113.51 ± 00.14^##^
	Inf.Chit	103.62 ± 0.05^#^	92.46 ± 0.14^#^	115.58 ± 0.05^##^
	Uni.Chit	103.44 ± 0.23^#^	92.45 ± 0.09^#^	134.92 ± 0.62^##^
	N.C	5.33 ± 0.06	4.56 ± 0.03	4.52 ± 0.04
	Inf.Unt	5.61 ± 0.41	5.71 ± 0.06^*^	5.85 ± 0.03^**^
Total protein (g/dl)	Inf.Par	5.48 ± 0.51	4.79 ± 0.03^#^	4.18 ± 0.02^#^
	Inf.Chit	5.50 ± 0.49	4.67 ± 0.07^#^	4.24 ± 0.03^#^
	Uni.Chit	5.45 ± 0.1	4.51 ± 0.02^#^	4.25 ± 0.06^#^
	N.C	22.87 ± 0.05	22.49 ± 0.07	31.25 ± 0.9
	Inf.Unt	32.32 ± 0.05^**^	35.98 ± 0.06^**^	25.32 ± 0.05^**^
Urea (mg/dl)	Inf.Par	21.88 ± 0.52^##^	23.74 ± 0.08^##^	33.23 ± 0.17^#^
	Inf.Chit	21.95 ± 0.6^##^	23.50 ± 0.4^##^	34.22 ± 0.13^##^
	Uni.Chit	22.21 ± 0.16^##^	22.62 ± 0.52^##^	33.90 ± 0.06^#^

ALP, alkaline phosphatase; ALT, alanine aminotransferase; AST, aspartate aminotransferase; N.C, normal control; Inf.Unt, infected + untreated; Inf.Par, infected + paromomycin; Inf.Chit, infected + chitosan; Uni.Chit, uninfected + chitosan. The above data presented as means ± SD of three independent tests. * and ** indicate significant differences at p < 0.05 and p < 0.01, N.C group compared with Inf.Unt group; ^#^ and ^##^ indicate significant differences at p < 0.05 and p < 0.01, Inf.Unt group compared with Inf.Par, Inf.Chit, and Uni.Chit groups.

### Chitosan Regulated Intestinal Cytokine Levels and Mediated TLR4/STAT1 Signaling Regulation in Mice Infected With *C. parvum*

**Figure 2:**
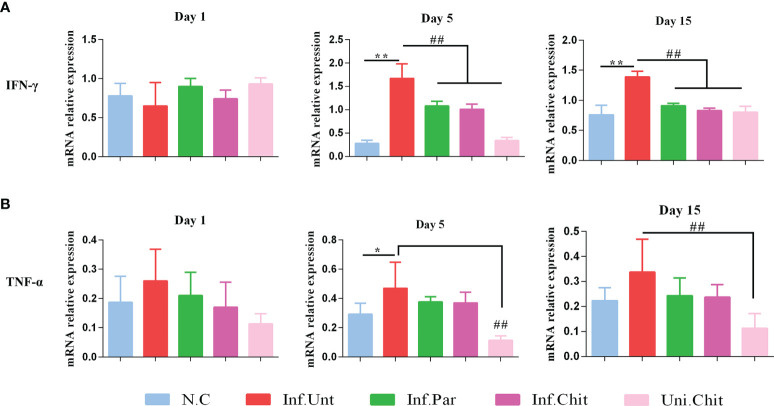
Chitosan supplementation regulated the expression level of inflammatory cytokines **(A)** IFN-γ and **(B)** TNF-α in C57BL/6 mice challenged with *C. parvum*. The above data presented as means ± SD of three independent tests. Significant differences among N.C vs. Inf.Unt groups are indicated by *p < 0.05, **p < 0.01. Significant differences between Inf.Unt vs. Inf.Par, Inf.Chit, and Uni.Chit are indicated by ^##^p < 0.01.

To quantify the mechanisms of chitosan involved in the progression of cryptosporidiosis, mRNA expression of intestinal cytokines including TNF-α and IFN-γ was examined (**Figures 2A, B** **)**. Compared with mice from the normal group, mice challenged with *C. parvum* in all other groups at D1PI showed no significant changes in cytokine mRNA expression including TNF-α and IFN-γ in ileum tissues. However, on day 5 and 15 post-infection, IFN-γ in mice of NC, Inf.Par, Inf.Chit, and Uni.Chit groups showed a significant reduction (*p <* 0.01) compared to the untreated group of infected mice (**Figures 2A, B** **)**. On the other hand, TNF-α on day 5 and 15 post-infection in mice of the Inf.Unt group showed no significant difference with Inf.Par and Inf.Chit groups, but on both day 5 and 15 post-infection, the expression was significantly reduced in mice of the Uni.Chit group (*p >* 0.01).

**Figure 3:**
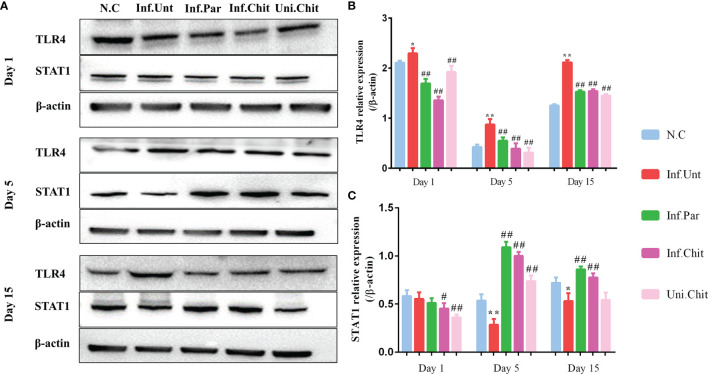
Effects of chitosan on the induction of the TLR4 and STAT1 signaling pathways. **(A)** Western blot detection of TLR4 and STAT1 expression in intestinal tissues. **(B, C)** The relative expression of TLR4 and STAT1 protein in each group. All values are indicated as the mean ± SEM of three independent tests. * and ** indicate significant differences at *p <* 0.05 and *p <* 0.01, Inf.Unt group compared with N.C group; ^#^ and ^##^ indicate significant differences at *p <* 0.05 and *p <* 0.01, Inf.Unt group compared with Inf.Par, Inf.Chit, and Uni.Chit groups. The experiment was repeated three times with similar results.

Meanwhile, chitosan consumption suppressed the induction of the TLR4 signaling pathway in mice. As shown in **Figures 3A, B**, the relative protein level of TLR4 increased dramatically (*p <* 0.01) in the mice of the Inf.Unt group compared with the other groups (**Figures 3A, B** **)** at day 1, 5, and 15 post-infection. Moreover, STAT1 signaling, which is an important regulator of IFN-γ signaling, was significantly blocked (*p <* 0.01) by *C. parvum* in the mice of the Inf.Unt group at day 5 and 15 post-infection compared with the NC, Inf.Par, Inf.Chit, and Uni.Chit groups (**Figures 3A, C** **)**.

### Chitosan Supplementation Prevented the Reduction of Bacterial Diversity Induced by *C. parvum*

**Figure 4:**
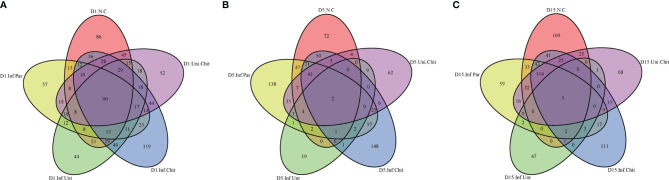
Unique and shared OTUs analysis of the different samples. Venn diagram explaining the overlap in gut microbiota ASVs/OUTs between the N.C, Inf.Unt, Inf.Par, Inf.Chit, and Uni.Chit groups. **(A)** Day 1 post-infection, **(B)** day 5 post-infection, and **(C)** day 15 post-infection total richness of OTUs shared between different groups.

To estimate the effect of chitosan on the gut microbial populations of mice infected with *C. parvum*, sequence reads were performed for each sample. Based on 99% sequence similarity, all sequences were clustered into ASVs/OTUs and assigned to 1,435 OTUs after quality filtering, denoise, and de-chimerism. The richness of OTUs between groups at day 1, 5 and 15 post-infection was compared. During the comparison at D1PI, 90 of 358 (>25.1% of total OTUs) were shared between groups (**Figure 4A**). On D5PI, the day of peak *C. parvum* infection, only 2 (0.46%) out of 439 OTUs were shared (**Figure 4B**), and on D15PI, 5 (1.27%) out of 394 OTUs were shared between N.C, Inf.Unt, Inf.Par, Inf.Chit, and Uni.Chit groups (**Figure 4C**).

**Figure 5:**
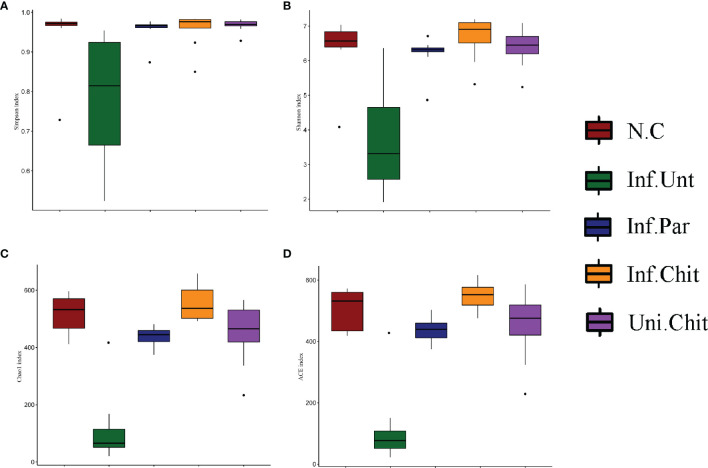
Alpha diversity analysis of gut microbiota in *C. parvum* infected mice following chitosan interventions. **(A, B)** Bacterial diversity **(C, D)** Community abundance.

Species accumulation curve (**Figure S1A**) and rarefaction curve (**Figure S1B**) displayed that the samples size, gut microbiota, and sequencing depth were adequate and met the standards for further investigation. Alpha diversity indices (Simpson and Shannon) and bacterial richness estimators (Chao1 and Ace index) were compared (**Figure 5**). As shown in **Figures 5A, B**, *C. parvum*-infected mice fed chitosan diet had higher bacterial diversity (Simpson and Shannon) than mice in the Inf.Unt group (*p >* 0.05). For community abundance indices (Chao1 and Ace), the value in the Inf.Unt group was significantly lower than in the N.C, Inf.Par, Inf.Chit, and Uni.Chit groups (*p <* 0.05) (**Figures 5C, D** **)**. Overall, these data suggested that mice infected with *C. parvum* had lower bacterial diversity, whereas supplemental chitosan increased gut microbial diversity.

**Figure 6:**
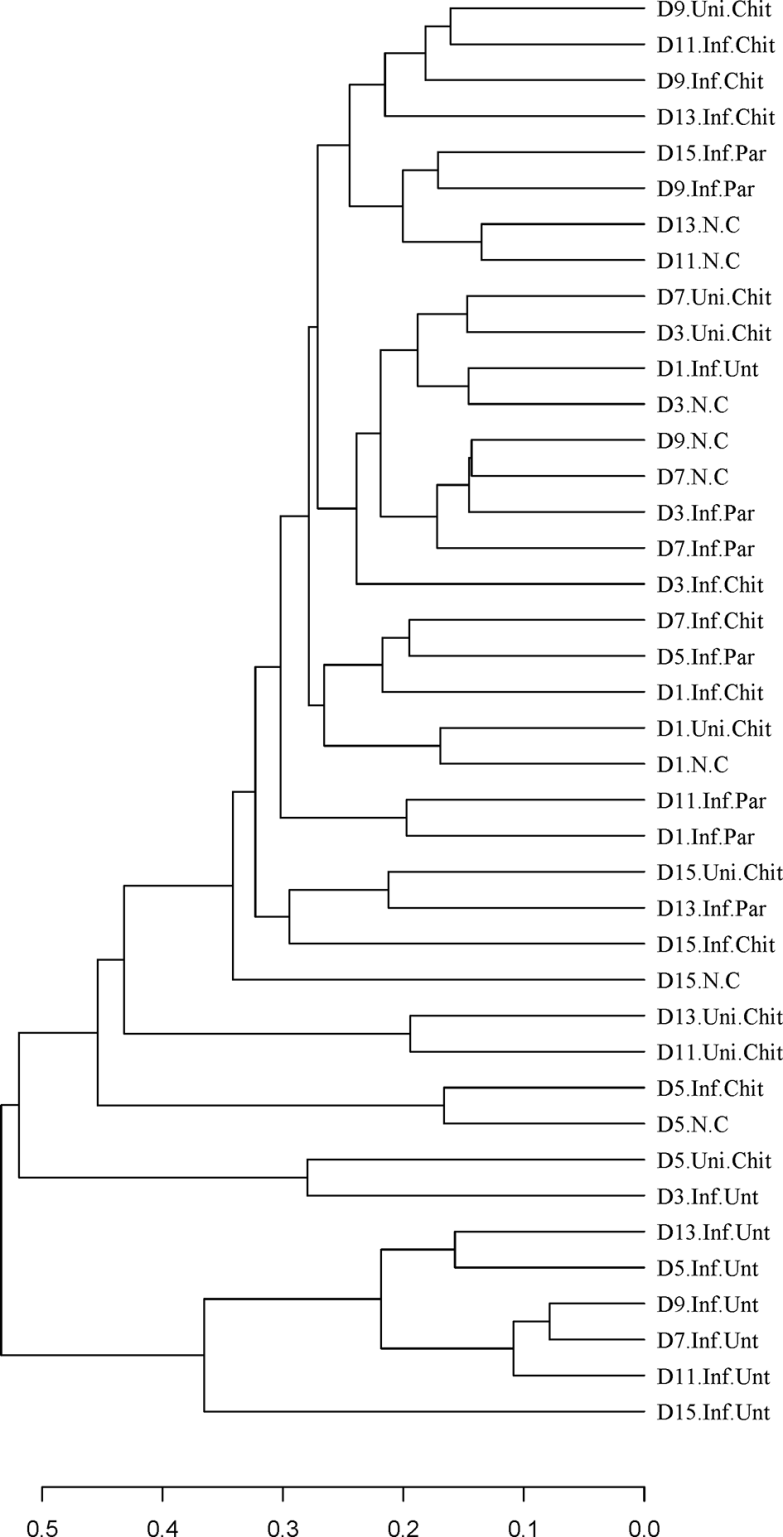
Differences in beta diversity (UPGMA weighted UniFrac distance) between N.C, Inf.Unt, Inf.Par, Inf.Chit and Uni.Chit groups at different DPI. Note: The samples are clustered according to the similarity between each other. The shorter the branch length between samples, the more similar the two samples.

Beta diversity analysis was used to confirm the differences and similarities in gut microbial community composition between groups (**Figure 6**). Hierarchical clustering analysis based on weighted UniFrac unweighted pair group method with arithmetic mean (UPGMA) confirmed that the gut microbiota of the Inf.Unt group showed high similarity and different clustering of microbial composition for each group during the different DPIs (**Figure 6**).

### Chitosan Supplementation Modulated Microbial Community Structure in Immunosuppressed Mice Infected With *C. parvum*

**Figure 7:**
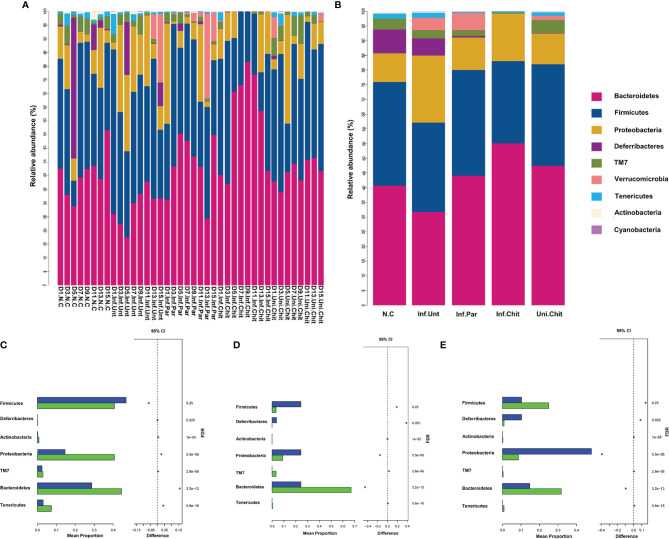
Chitosan supplementation modulated gut microbial community at phylum level in mice infected with *C*. *parvum*. **(A)** Taxonomic distributions of the gut microbiota at phylum level in all samples. **(B)** Average gut microbial composition of the phylum level in five groups. **(C**–**E)** Relative abundance of the gut microbial community between Inf.Unt vs. Inf.Chit groups at phylum level. **(C)** Day 1, Inf.Unt_Vs Inf.Chit. **(D)** Day 5, Inf.Unt_Vs Inf.Chit. **(E)** Day 15, Inf.Unt_Vs Inf.Chit. Blue color indicates Inf.Unt, while green color represents Inf.Chit group. The figure shows the difference ratio of functional abundance in 95% confidence interval; the right value is the p-value and if *p <* 0.05, designating significant difference.

At the phylum level, the histogram (**Figure 7A**) shows the microbial community structure in the gut and describes the microbial species and their relative abundance during the different DPI. Bacteroidetes, Firmicutes, Proteobacteria, and Deferribacteres were the dominant phyla of the total sequence reads (**Figure 7B**). At D1PI, the relative abundance of Bacteroidetes was low in the Inf.Unt group, but Firmicutes was higher than in the Inf.Chit group (*p >* 0.05). In addition, Proteobacteria and Tenericutes were significantly decreased in the Inf.Unt group compared with the Inf.Chit group (*p <* 0.05) (**Figure 7C**). At D5PI, the relative abundance of Bacteroidetes was decreased, and that of Proteobacteria was increased in the Inf.Unt group compared with the Inf.Chit group, but the data were not significant (*p >* 0.05). On the same day, Defferribacteres and Firmicutes showed significant decrease in Inf.chit group compared with the Inf.Unt group (*p <* 0.05) (**Figure 7D**). Similarly, on D15PI, Defferribacteres were significantly decreased in the Inf.Chit group compared to the Inf.Unt group (*p <* 0.05) as shown by STAMP analysis in **Figure 7E**.

**Figure 8:**
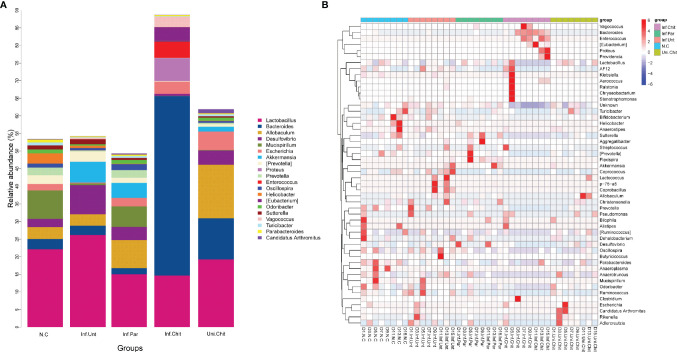
Chitosan treatment modulated gut microbiota at the genus level in mice infected with *C parvum*. **(A)** Histogram represents the relative abundance of the top 20 microbial genera in the feces of mice from the five groups. **(B)** Heatmap indicate the top 50 microbial genera based on UPGMA clustering method and Bray–Curtis distance metric for *C parvum*-infected mice given different treatments at different DPIs.

At the genus level, the microbial populations of the 20 major genera were identified in all samples (**Figure 8**). Of these, *Lactobacillus*, *Bacteroides*, *Allobaculum*, *Desulfovibrio*, *Mucispirrillum*, *Escherichia*, and *Akkermansia* dominated in different groups (**Figure 8A**). The genus *Bacteroides* was highly dominated in the Inf.Chit compared to Inf.Unt and other groups (**Figure 8A**). At D1PI, STAMP analysis (**Figure S2A**) and heat map (**Figure 8B**) showed that the relative abundance of *Lactobacillus*, *Desulfovibrio*, and *Escherichia* were lower in the Inf.Unt group compared to that in the Inf.Chit group; however, no significant differences were observed between the two groups (*p >* 0.01). On the same day, *Bifidobacterium* and *Clostridium* were highly significantly increased in the Inf.Unt group compared to that in the Inf.Chit group (*p <* 0.01). At D5PI, the peak of infection, the relative abundance of *Lactobacillus* and *Desulfovibrio* decreased, whereas *Escherichia* increased in the Inf.Unt group compared with that in the Inf.Chit group (*p >* 0.01) (**Figure 8B**; **Figure S2B**). Some genera such as *Mucispirillum*, *Ruminococcus*, and *AF12* were highly significantly increased (*p <* 0.01) in the Inf.Unt group compared with the Inf.Chit group (**Figure 8B**). Similarly, on D15PI, the last day of the experimental period D15PI, the abundance of *Bifidobacterium*, *AF12*, and *Clostridium* were highly significantly increased in the Inf.Unt group compared with the Inf.Chit group (*p <* 0.01). On the same day, the relative abundance of some genera such as *Lactobacillus*, *Desulfovibrio*, Oscillospira, and *Ruminococcus* was decreased in the Inf.Chit group compared with the Inf.Unt group (**Figures 8B**, **S2C**). From D5PI, when the infection level reached the peak, the abundance of *Bacteroides* and the phylum Bacteroidetes were continuously higher in the Inf.Chit group compared with the Inf.Unt group (**Figures 7**, **8**).

## Discussion

The major problem associated with cryptosporidiosis is the lack of an effective means of treating and preventing this important zoonotic disease. In a previous study, more than 200 substances were tested to find the best possible therapeutic agent to treat cryptosporidiosis. However, none of them could successfully and completely eliminate *C. parvum* infection or control the clinical findings (30). In addition, some drugs have been shown to reduce oocyst shedding and pathogen detection in susceptible hosts (31). Studies have reported both *in vivo* and *in vitro* treatment effects of halofuginone lactate, ginsenoside-Rh2, and curcurbitacin-B against *Cryptosporidium* (32, 33). However, these compounds showed adverse effects and failed to eradicate the infection. In addition, some antibiotics such as azithromycin, sulfaquinoxaline, and paromomycin have been shown to reduce the level of infection and stop the shedding of *C. parvum* oocysts, but there is a risk of antibiotic residues remaining. Recently, several studies have shown the protective effect and antimicrobial activity of chitosan and its derivatives against various infectious diseases including *C. parvum* (17, 18, 34). In addition, chitosan has been used as an excipient for microparticle systems and has been reported that it can provide a physical barrier against *C. parvum* infection in a neonatal mouse model (35). As a result, it has been quantified that chitosan has a promising future in preventive and therapeutic applications against *C. parvum* infections, but the mechanisms of action need further clarification. In this study, we have established *C. parvum* infection in juvenile mice immunosuppressed with dexamethasone. The easy-to-use and low-cost immunosuppression experimental model used in our study showed 100% survival of the mice, which allowed us to study the acute course of *Cryptosporidium* infection. The mice model used in our study also revealed that it would be an appropriate model for studying various aspects of *Cryptosporidium* infection, particularly innate immune responses. Same as the above reports, in this study, the excretion of *C. parvum* oocysts significantly decreased in the infected group treated with chitosan compared with the infected group receiving no treatment (*p <* 0.01). Between the groups, a significant reduction at peak infection D5PI was observed in Inf.Par and Inf.Chit compared to Inf.Unt group (*p <* 0.01). Overall, compared with the Inf.Unt group during the experimental period, a significant decrease in oocysts excretion of 58.82% and 75.54% was observed in Inf.Par and Inf.Chit groups, respectively. Furthermore, BW gain was also significantly (*p <* 0.01) decreased followed by loss of appetite in the Inf.Unt group compared with other groups during the experimental period.

A previous study has shown that an elevated level of AST in serum indicates damage to the mitochondria of the liver tissue (36). Moreover, elevated levels of ALP and ALT are used as signs of hepatic cellular damage and hepatobiliary diseases (37). In the present study, elevated levels of the enzymes AST, ALT, and ALP in the Inf.Unt mouse group indicated that *C. parvum* causes damage to hepatocyte in juvenile mice. In addition, *C. parvum*-infected mice that did not receive treatment were found to have a significant decrease in albumin and increased total protein (*p <* 0.05) compared with Inf.chit and the other groups. Since the liver is responsible for the synthesis of albumin, the decreasing albumin level might contribute to hepatocellular damage (38). A previous study reported that albumin was not significantly affected, whereas globulin decreased significantly during *Cryptosporidium* infection (39). In our study, the enzymes AST, ALT, ALP, albumin, urea, creatinine, and glucose were restored to normal levels in the serum of Inf.Chit-treated mice, suggesting that chitosan has a suppressive effect against *C. parvum* infection. Furthermore, this suggests that chitosan greatly attenuates the deleterious effects of *C. parvum* by reducing or increasing serum concentrations of AST, ALT, ALP, albumin, urea, creatinine, and glucose to normal levels during *C. parvum* infection.

The induction of cytokines such as IL-1, IL-2, IL-4, IL-5, IL-6, IL-8, IL-15, IFN-γ, and TNF-α has been previously reported in the context of *C. parvum* infection (40). Studies have shown that the important cytokines IFN-γ and TNF-α play a critical role in host resistance to parasite invasion (41, 42). IFN-γ has been shown to be important in host resistance to *C. parvum* infection in humans and mice. A previous study reported that IFN-γ knockout mice were heavily infected with *C. parvum* during the experimental period (43). Moreover, IFN-γ along with TNF-α contributes to macrophages releasing higher levels of IL-12 in the initial and early stages of *Cryptosporidium* infection to control parasite replication (44). In our study, *C. parvum* significantly (*p <* 0.01) increased the mRNA expression level of IFNv-γ in the infected group receiving no treatment compared with the infected group supplemented with chitosan or paromomycin during D5PI, the peak of infection, and D15PI. On the other hand, TNF-α showed a non-significant decrease in Inf.Chit, Inf.Par, and N.C group at D5PI and D15PI compared to that in the Inf.Unt group, whereas the levels decreased significantly in the Uni.Chit group. In agreement with a previous study, innate immune responses during *C. parvum* showed higher expression of immune mediators including TNF-α, IFN-γ, and IL-6 by Th1, which mainly defend against *C. parvum* and intracellular infections (45). Thus, chitosan supplementation could regulate cytokines and stimulate immune cells that further enhance host resistance to *C. parvum* infection. Our results also suggest that chitosan protects against *C. parvum* infection by inhibiting the expression of inflammatory cytokines.

Toll-like receptors (TLRs) play an important role in the activation of innate immunity and pathogen recognition (46, 47). Previous studies have shown that the expression of TLR4 increases during *C. parvum* infection (48). TLR4 could induce dendritic cells to recognize *C. parvum* and induce Th1 immune response to control *C. parvum* infection (49). In addition, TLR4 is required for an effective IFN-γ response, but it is likely that other components of host innate immunity are also required and important for the elimination of *C. parvum* infection based on TLR4 signaling (49). In this study, we found that chitosan significantly suppressed the induction of relative protein expression of TLR4 in the juvenile mice infected with *C. parvum*. In addition, the group of infected mice that received no drug treatment showed higher TLR4 protein expression compared with the other groups at D5PI and D15PI. These results suggest that TLR4-mediated response is critical for effective clearance of *C. parvum* infection in mice and that chitosan could downregulate TLR4 signaling to control *C. parvum* infection. On the other hand, STAT1, an important regulator of IFN-γ signaling, is blocked by *C. parvum*, as proposed by previous studies (50, 51), and interferon regulatory factor-1 (IRF-1), which regulates various innate and adaptive immune responses, have been shown to reduce during *C. parvum* infection (52). At the cellular level, IFN-γ mediates its action by binding to IFN-γR, leading to the activation of STAT1 (53). In our study, chitosan supplementation resulted in significant upregulation of STAT1 signaling. At D5PI and D15PI, the relative protein levels were blocked in the *C. parvum*-infected group that did not receive drug treatment; however, protein levels were significantly increased in the infected group supplemented with chitosan. In investigating the possible mechanism for these results, we propose that *C. parvum* infection caused depletion of STAT1 protein, an important transcription factor in IFN-γ signaling, while chitosan upregulated STAT1 signaling to regulate IFN-γ signaling.

Various diseases and gut health are associated with the gut microbiota, including the metabolism and digestion of food (54). Previous studies have reported various biological and health-related functions of chitosan, including antimicrobial activities and immune stimulation (55). On the other hand, stable and healthy gut microbiota plays an important role in modulating host immunity, inhibiting the development of intestinal diseases, and resistance to various pathogens (56, 57). Diet, infection level, stress, and various drugs are known to affect the gut microbiota (6, 58). Therefore, we investigated the gut microbial community of juvenile mice infected with *C. parvum* and supplemented with chitosan using 16S rRNA gene sequencing analysis. Microbial diversity indices were decreased but not significantly affected by *C. parvum* or supplemental chitosan. However, microbial community richness in the *C. parvum*-infected group that received no drug treatment decreased significantly compared to the infected group supplemented with chitosan. The increased bacterial richness and diversity are important for host health because these indices contribute significantly to unique functions and various metabolic traits that lead to a healthy environment and reduce the possibility of proliferation of pathogenic gut bacteria (59). This could be due to the fact that *C. parvum* colonizing the intestinal epithelium has displaced the local bacterial flora, resulting in a decrease in microbial diversity in the Inf.Unt group. Microbial community structure (beta diversity) showed significant differences between groups in our study, suggesting that chitosan supplementation shaped the structure of the gut microbiota in juvenile mice infected with *C. parvum*.

In our study, the dominant phyla were Bacteroidetes, Firmicutes, Proteobacteria, and Deferribacteres in all groups. The relative abundance of Proteobacteria was significantly lower in the *C. parvum*-infected mice supplemented with chitosan than in the infected mice that did not receive drug treatment. This is consistent with a previous study conducted on rats infected with *E. coli* and treated with chitosan-chelated zinc (60). Proteobacteria belong to the Gram-negative bacteria, and increased abundance of members of this phylum has been associated with various diseases, including inflammatory bowel disease and metabolic disorders (61). Therefore, the decrease in Proteobacteria in the chitosan-treated *C. parvum*-infected group might be related to the intestinal health of the mice. On the other hand, Bacteroidetes are tremendous utilizers of plant polysaccharides and some other refractory nitrogen and organic carbon sources. In a study, Bacteroidetes were reported to help in the prevention of diarrhea, as members of this phylum are associated with the gut immune system, indicating a close relationship between gut immune maturation and neonatal microbiota colonization after weaning (62). In the present study, the abundance of Bacteroidetes was continuously high after D5PI in the infected group of mice treated with chitosan (**Figure 7**). These results suggest that Bacteroidetes have a strong effect during the onset of diarrhea and that a high-carbohydrate diet, such as the administration of chitosan, stimulates and increases the amount of Bacteroidetes to help break down carbohydrates and reduce diarrhea by improving the gut immune system. Further studies are needed to determine the relationship between the abundance of Bacteroidetes and acute and chronic diarrhea caused by *C. parvum*.

At the genus level, chitosan supplementation significantly increased the relative abundance of *Bacteroides*, *Proteus*, and *Enterococcus* compared with the *C. parvum*-infected group that received no drug treatment. Moreover, the abundance of the important genus *Lactobacillus* was decreased at D5PI in the Inf.Unt group compared with the Inf.Chit group. Chitosan oligosaccharides are the degradation product of amino polysaccharides and important substrates for gut microbes, so they can change the structure of gut microbiota (63). Similar to the phylum Bacteroidetes, the genus *Bacteroides* degrades complex carbohydrates from the diet and modulates gene expression and immune maturation (64). Moreover, Bacteroidetes can promote intestinal peristalsis by increasing the expression of γ-actin, enteric γ-aminobutyric acid, and vesicle-associated protein-33 (64). Therefore, in our study, the higher abundance of *Bacteroides* similar to the Bacteroidetes phylum due to chitosan supplementation in the *C. parvum*-infected mice could be a possible mechanism that reduced the excretion of *C. parvum* oocysts (**Figures 7**, **8**). Moreover, in the present study, the total abundance of *Desulfovibrio* decreased in the infected group supplemented with chitosan compared to the infected group receiving no treatment. In the intestine, *Desulfovibrio* is the major sulfate-reducing microbial species. One study reported that an increase in the abundance of *Desulfovibrio* significantly increased the possibility of ulcerative colitis in the colon at numerous stages (65). Here, a lower abundance of *Desulfovibrio* in the Inf.Chit group showed that chitosan alleviated intestinal inflammation in the *C. parvum*-infected mice group compared to the Inf.Unt group. On the other hand, the incidence of enterococci was higher in the *C. parvum*-infected group treated with chitosan than in the *C. parvum*-infected-untreated group. *Enterococci*, which belong to the genus *Enterococcus*, are often studied as potential probiotic candidates. *Enterococcus* probiotics are mainly used for the prevention and treatment of antibiotic-induced diarrhea, irritable bowel syndrome, and chronic intestinal diseases (66). In addition, some *Enterococcus* members showed hypocholesterolemic, anticarcinogenic, immunostimulatory, and regulatory effects and functionally prevent dysbiosis (67). However, further studies are needed to prove the probiotic effect of chitosan in curbing diarrheal diseases recommended in the reference study (68).

Although our study suggests that chitosan-mediated protection by the gut microbiota plays a role in inhibiting *C. parvum* infection, there are likely other factors that contribute to the increased susceptibility of mice to *C. parvum* infection. We demonstrated that the protective effects of chitosan during *C. parvum* infection is associated with gut microbiota modulation, in particular with a higher abundance of Bacteroidetes*/Bacteroides*. In addition, chitosan reshaped the structure of the gut microbiota, which was critical for the production of metabolites in the gut and the integrity of the gut barrier. Moreover, several mechanisms are involved in the anti-*Cryptosporidium* effects of chitosan, including composition of the gut microbiota, suppression of cytokine storm, and restoration of normal cytokine levels, and up- and downregulation of STAT1/TLR4 signaling pathways. Our research findings point towards a potential application of chitosan in the treatment and prevention and rendering TLR4/STAT1 pathways a potential target for therapy aiming at reducing *C. parvum* infection.

## Data Availability Statement

The datasets presented in this study can be found in online repositories. The names of the repository/repositories and accession number(s) can be found below: https://www.ncbi.nlm.nih.gov/genbank/, accession IDs OK626596-OK626635.

## Ethics Statement

The animal study was reviewed and approved by Animal Ethical Committee of Shanghai Veterinary Research Institute, Chinese Academy of Agricultural Sciences (SV-20201231-03).

## Author Contributions

SR and ZC designed the project. SR and RM contributed significantly in lab work. SR and RM collected the samples. HG, YH, and XH helped analyzed the data. SR wrote the manuscript. HG and YH contributed to statistical analysis of data. ZC supervised the whole work. All authors contributed to the article and approved the submitted version.

## Funding

This study was supported in part by the Shanghai Agriculture Applied Technology Development Program, China (Grant No. 2019-02-08-00-08-F01151 and G20150110), Shanghai Science and Technology Commission Scientific Research Project (Grant No. 20140900400), Scientific Research Project of Special Training Program for Scientific and Technological Talents of Ethnic Minorities in Xinjiang (Grant No. 2020D03030), National Risk Assessment Project for Quality and Safety of Agricultural Products (Grant No. GJFP2019027), and National Key Research and Development Program of China (Grant No. 2017YFD0500401).

## Conflict of Interest

The authors declare that the research was conducted in the absence of any commercial or financial relationships that could be construed as a potential conflict of interest.

## Publisher’s Note

All claims expressed in this article are solely those of the authors and do not necessarily represent those of their affiliated organizations, or those of the publisher, the editors and the reviewers. Any product that may be evaluated in this article, or claim that may be made by its manufacturer, is not guaranteed or endorsed by the publisher.
