# Supplementary material for: Chitosan Protects Immunosuppressed Mice Against Cryptosporidium parvum Infection Through TLR4/STAT1 Signaling Pathways and Gut Microbiota Modulation
Source: Front Immunol. 2022 Jan 14;12:784683. doi: 10.3389/fimmu.2021.784683 (PMC8795679; doi:10.3389/fimmu.2021.784683)
Supplement: Supplementary file 1 [file DataSheet_1.docx]

***Supplementary Material***

**Chitosan Protects Immunosuppressed Mice against *Cryptosporidium parvum* Infection through TLR4/STAT1 Signaling Pathways and Gut Microbiota Modulation**

***Sajid Ur Rahman, Haiyan Gong, Rongsheng Mi, Yan Huang, Xiangan Han, Zhaoguo Chen^*^***

*Key Laboratory of Animal Parasitology of Ministry of Agriculture, Laboratory of Quality and Safety Risk Assessment for Animal Products on Biohazards (Shanghai) of Ministry of Agriculture, Shanghai Veterinary Research Institute, Chinese Academy of Agricultural Sciences, Shanghai 200241, China*

****Corresponding author:*** *Zhaoguo Chen, Key Laboratory of Animal Parasitology of Ministry of Agriculture, Laboratory of Quality and Safety Risk Assessment for Animal Products on Biohazards (Shanghai) of Ministry of Agriculture, Shanghai Veterinary Research Institute, Chinese Academy of Agricultural Sciences, Shanghai 200241, China*

*Email address:* [*zhaoguochen@shvri.ac.cn*](file:///C:\Users\user\Desktop\文章\zhaoguochen@shvri.ac.cn) *(ZC)*

*Tel.: +86 21 34293157*

*Fax: +86 21 54081818*

**Running Title**

Chitosan Mitigates *Cryptosporidium parvum* Infection

**TABLE S1:** Primer sequences used for qPCR

| **Gene** | **Primer Sequences** | **GenBank accession number** | **Product size (bp)** |
| --- | --- | --- | --- |
| *IFN-γ* | Forward: 5'-ATGAACGCTACACACTGCATC-3'  Reverse: 5'-CCATCCTTTTGCCAGTTCCTC-3' | NC_000076.7 | 182 |
| *TNF-α* | Forward:5'-GGAACACGTCGTGGGATAATG-3' Reverse: 5'-GGCAGACTTTGGATGCTTCTT-3' | NP_038721.1 | 235 |
| *GAPDH* | Forward: 5'-TTCTTGTGCAGTGCCAGCCTC-3'  Reverse: 5'-CCGGCATCGAAGGTGGAAGA-3' | NM_008084.3 | 922 |

**Table S2: Changes in body weight of *C. parvum* infected mice following chitosan treatment.**

| **Groups** | **DBI** | **Day 1** | **Day 4** | **Day 7** | **Day 10** | **Day 13** | **Day 15** |
| --- | --- | --- | --- | --- | --- | --- | --- |
| N.C | 9.18 ± 0.61 | 9.84 ± 0.03 | 11.70 ± 0.13 | 12.30 ± 1.24 | 14.23 ± 1.12 | 15.04 ± 1.40 | 17.60 ± 1.14 |
| Inf.Unt | 9.01 ± 0.55 | 9.67 ± 0.52 | 10.26 ± 0.54^**^ | 10.21 ± 0.72^**^ | 11.77 ± 1.20^**^ | 12.45 ± 0.10^**^ | 14.27 ± 0.85^**^ |
| Inf.Par | 8.86 ± 1.5 | 9.19 ± 0.02^#^ | 12.55 ± 0.14^##^ | 13.24 ± 1.20^##^ | 14.60 ± 0.27^##^ | 15.12 ± 0.74^##^ | 16.37 ± 0.16 ^##^ |
| Inf.Chit | 8.83 ± 1.41 | 9.49 ± 0.08 | 11.41 ± 1.12^##^ | 12.19 ± 1.16^##^ | 13.30 ± 1.16^##^ | 15.10 ± 0.36^##^ | 16.55 ± 1.91^##^ |
| Uni.Chit | 9.32 ± 0.54 | 9.66 ± 0.04 | 12.55 ± 0.30^##^ | 14.68 ± 0.71^##^ | 14.85 ± 0.10^##^ | 15.26 ± 0.41^##^ | 16.40 ± 1.30^##^ |

DBI, Day before infection; N.C, Normal control; Inf.Unt, Infected+Untreated; Inf.Par, Infected+Paromomycin; Inf.Chit, Infected+Chitosan; Uni.Chit, Uninfected+Chitosan. The above data presented as means ± SD of three independent tests. Note: * and ** indicate significant differences at *p <* 0.05 and *p <* 0.01 compared N.C group with Inf.Unt group; # and ## indicate significant differences at *p <* 0.05 and *P <* 0.01 compared Inf.Unt group with Inf.Par, Inf.Chit, and Uni.Chit groups.

**
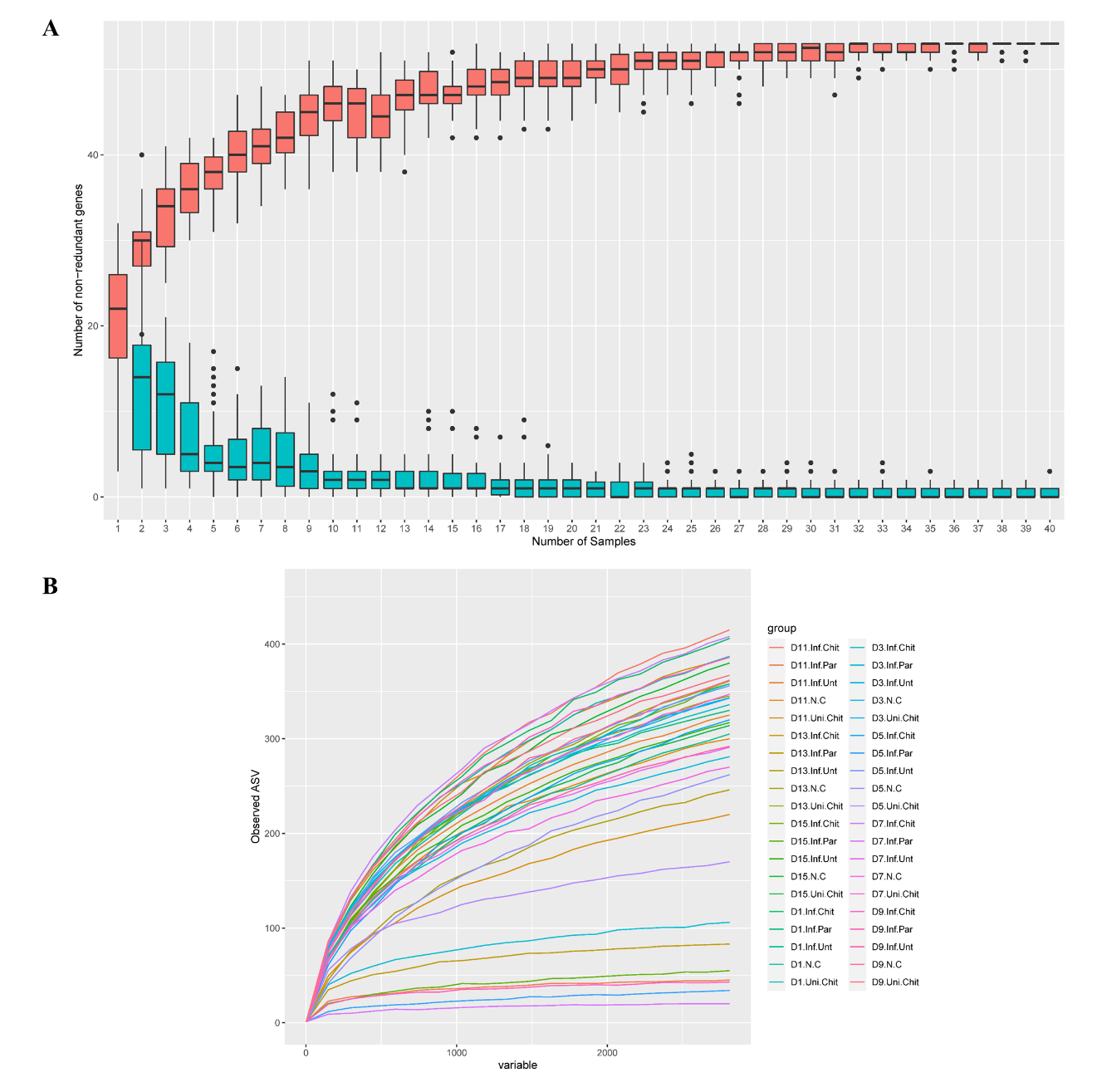
**

**FIGURE S1:** Species accumulation curve based on the sequenced number (**A**). Note: The abscissa is the number of sequenced samples randomly selected, and the ordinate is the number of ASV/OTUs obtained by clustering. Each curve represents a group marked with a different color **(B)** Rarefaction curve of microbiota based cumulative curve of horizontal species of genus. Note: The abscissa represents the sample size; the ordinate represents the number of species after sampling; the red box line forms the cumulative curve; the green box line forms the common volume curve.

**
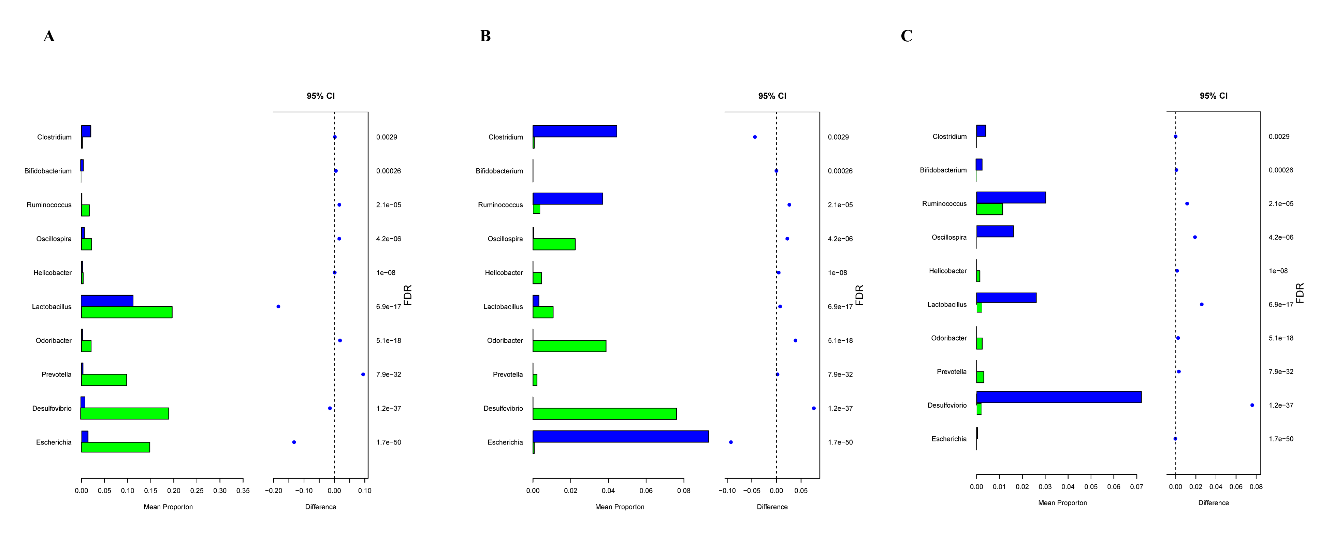
**

**FIGURE S2:** STAMP analysis indicating gut microbial community differences between Infected.Untreated and Infected.Chitosan treated groups at the genus level. The figure show top 10 species with signifcant differences. (A) Day 1. Inf.Unt_Vs Inf.Chit (B) Day 5. Inf.Unt_Vs Inf.Chit (C) Day 15. Inf.Unt_Vs Inf.Chit. The figure shows the difference ratio of functional abundance within 95% confidence interval, the right value is the P value, *P <* 0.01, showing significant difference between the two groups.
